# Efficacy of zirconium oxide nanoparticles coated on stainless steel and nickel titanium wires in orthodontic treatment

**DOI:** 10.6026/97320630017760

**Published:** 2021-08-31

**Authors:** Abirami Selvaraj, Ashwin Mathew George, S Rajeshkumar

**Affiliations:** 1 Department of Orthodontics and Dentofacial Orthopaedics, Saveetha Dental College and Hospitals, Saveetha Institute of Medical and Technical Sciences (SIMATS), Saveetha University, Chennai-77, India; 2 Nanobiomedicine Lab, Department of Pharmacology, Saveetha Dental College and Hospitals, Saveetha Institute of Medical and Technical Science, Saveetha University, Chennai-77, India

**Keywords:** clove and cardamom, zirconium oxide nanoparticles, antibacterial, anti inflammatory, cytotoxicity, TEM

## Abstract

It is of particular intrigue to synthesize, analyze anti-bacterial, anti-inflammatory activity, cytotoxicity effect of clove and cardamom reinforced zirconium oxide nanoparticles to coat the orthodontic archwires and study its ramifications. Characterization of nanoparticles was done using Transmission electron microscopic analysis (TEM). Antimicrobial activity was assessed using agar well diffusion method. Cytotoxic effect was assessed using Brine Shrimp Assay. Anti-inflammatory activity was completed using Bovine Serum Albumin (BSA). A Digital magnetic stirrer with a hot plate was used to coat orthodontic arch wires such as NiTi and SS. TEM spherical shape was of size 5 -20 nm. Minimal cytotoxicity was observed at 50 µL. Anti-inflammatory property was fair. Antimicrobial activity against Lactobacillus species, streptococcus mutans staphylococcus aureus and Candida albicans was recorded. NiTi and SS showed a colour shift from silver to orange red with a uniform surface coating on wires. Thus, green synthesized zirconium oxide nanoparticles have potent antimicrobial, anti-inflammatory properties with minimal cytotoxicity for further consideration as nano-coatings on orthodontic archwires such as NiTi and Stainless Steel.

## Background:

One of the greatest challenges in orthodontic biomechanics is to minimize the friction, which occurs at a number of points along the archwire. The variables, which cause friction, are mainly between the fixed appliance components such as the bracket, the archwire, the ligation system and biological factors [1]. As a bracket slides along an archwire, friction is only a small part of the resistance to tooth movement known as resistance to sliding (RS) [2, 3]. The three components of RS: (1) Friction, static or kinetic (FR) (contact between wire and bracket surfaces), (2) binding (BI), which happens when the tooth tips or the wire bends until the wire contacts the bracket edges, and (3) notching (NO), which occurs when the wire deforms permanently at the wire-bracket corner interface [3]. As a result, the maximum of 40 to 60% orthodontic forces is required to move a tooth as well as overcome friction in bracket-archwire interface. Concurrently, it raises the risk of root resorption and anchorage loss [4-6].

Friction is defined as the force that retards the movement when two objects move tangentially against one another [7]. As a result, two force components arise: frictional force (directed in a tangential direction to the contacting surfaces) and normal force (directed perpendicular to the contacting surfaces) [8]. Friction is proportional to normal force and is defined by the equation F = µN, where µ = the coefficient of friction [2].

Different methods have been proposed to counteract the frictional resistance such as Coating and modifying the surface of orthodontic metallic wires using various techniques and materials [9,10], methods of ligation [11] to address this issue [12]. Nanotechnology is a branch of science that studies nanomaterials with diameters ranging from 1 to 100 nanometers and has applications in dentistry, pharmaceuticals & bioengineering [13]. Nanotechnology is playing an essential role in almost every field of orthodontics, with one of the most important benefits being the reduction of friction by coating archwires and brackets. Besides that, nanoparticles outperform other orthodontic materials such as elastomeric ligatures, orthodontic adhesives, Temporary Anchorage Devices (TADs). Earlier application of nanotechnology started in the 1990s with regard to reduce the friction using metal dichalcogenide nanoparticles as solid lubricants in biomedical technology. Based on this concept, Samorodnitzky et al and Naveh et al [14] reported that coating of Nickel-Titanium (NiTi) wires with tungsten disulfide (WS2)  nanoparticles reduced friction. Stainless steel wires have also been coated with a composite of nickel-phosphorous and fullerene-like tungsten disulfide (WS2) nanoparticles as well as Cobalt (Co) and fullerene-like WS2 nanoparticles [15,16].

Considering the toxicity of WS2, nanoparticles using other metals such as Carbone Nitride (CNx) [17], ZnO [18,19], Inorganic fullerene like Molybdenum Disulfide nanoparticles [20], nanoceramics [21] etc., have been used. Recently, some studies have seen friction characteristics by using coated archwires with silver [22],zinc [16,^23^, [24] nanoparticles. However, the preparation of nanoparticles necessitates a large scale of laborious work, low efficiency, heavy metals, poor material conversion, and high-energy requirements [25]. Since synthetic stabilisers can contain hazardous byproducts, a green chemistry approach is being considered as an alternative. Green chemistry is emerging as an easy, cost-effective, repeatable, and environmentally acceptable method of producing stable metallic nanoparticles [26]. Syzygium aromaticum (clove) is valuable as a spice and has antimicrobial, antifungal, antioxidant, and anti-diabetic properties [27]. Cardamom (Elettaria cardamomum), a perennial aromatic plant that has antioxidant, anti-inflammatory, anti-cancer, and antimicrobial properties. Cardamom oil has been shown to have antibacterial activity against Streptococcus mutans in oral infections [28]. While clove and cardamom-mediated nanoparticles have been studied previously [29], none of the experiments have been used to synthesise zirconium oxide nanoparticles. Zirconium oxide has high refractive index, good optical and dielectric properties, high threshold of resistance, excellent biocompatibility, high corrosion resistance, and exhibit good adhesion onto metallic surfaces [30,31]. Different methods of coatings available such as thermal spray, physical and chemical vapour deposition, Electron- beam physical vapour deposition and ion implantation to improve the surface characteristics of the materials [32]. Therefore, it is of interest to synthesize, analyze the anti-bacterial, anti-inflammatory activity, cytotoxicity effect of clove and cardamom reinforced zirconium oxide nanoparticles to coat the orthodontic archwires.

## Materials & Methods:

### Materials and Preparation of plant extract:

**Figure 1:**
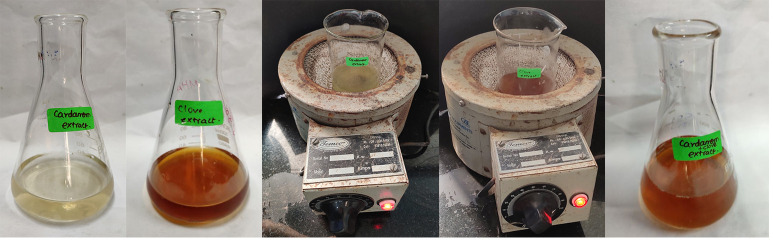
Preparation of plant extract: 0.5 g of dried powder of clove and cardamom was taken in 50 ml of distilled water and kept in a heating mantle at 60 -80 degree for 5 -10 min and red colored and light green colored solution was formed respectively. Filtration was done using Whatman filter paper no.1.30 ml of each filtrate was mixed together and boiled for 1 min and filtrate was stored.

50g of dried cardamom and clove were collected and ground to powder by using an electric mixer. 0.5 g dried clove and cardamom powder was mixed with 50 ml distilled water in a heating mantle and boiled at 60-80 degrees for 5-10 minutes, yielding a red and light green coloured solution, respectively. The clove and cardamom extract solution was cooled and filtered using Whatman No.1 filter paper. 30 mL of each filtrate was combined and heated for 1 minute, after which the filtrate was stored and utilized to synthesize zirconium oxide nanoparticles (Figure 1).

### Green synthesis of zirconium oxide nanoparticles:

**Figure 2:**
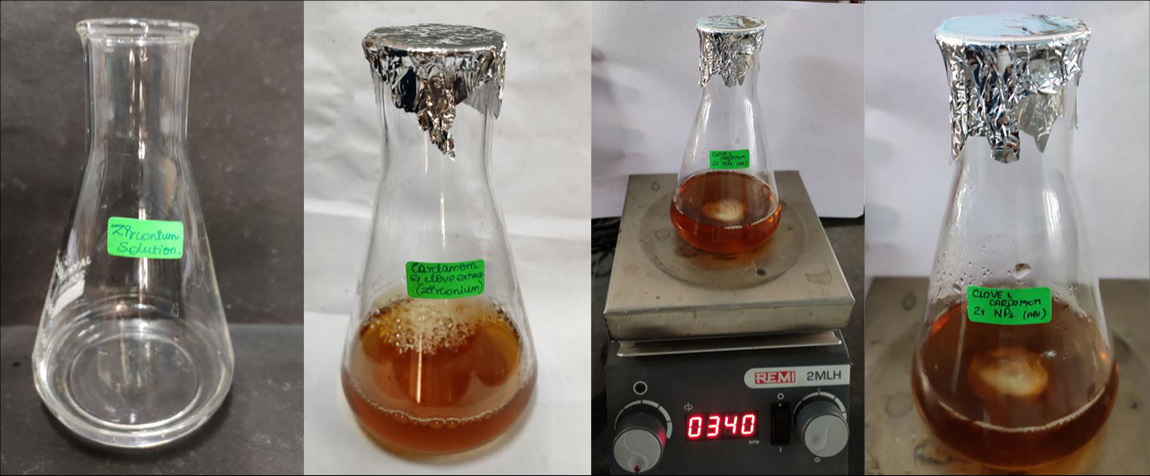
Preparation of ZrO2 nanoparticles and visual observation: 60 ml of cardamom and clove extract were added to 40 ml of zirconium precursor solution and mixture was continuously stirred using magnetic stirrer at 340–350°C and also kept overnight on an orbital shaker till color change was observed from light orange red colored to dark orange red colored solution indicating the formation of nano particles.

To make 0.01 N of (zrocl2.8H20) solution, 0.644 g salt of zirconium oxychloride octahydrate 98 percent was dissolved in 40 ml distilled water. 60 mL cardamom and clove extract were added to 40 mL zirconium precursor solution, and the mixture was continuously agitated at 340-350°C with a magnetic stirrer and stored overnight on an orbital shaker until colour change was seen (Figure 2). The colour change was monitored at an hourly interval for three days. At the end of the third day, the light orange red coloured solution had changed to a dark orange red coloured solution. UV spectroscopy was used to check for nanoparticle synthesis from the prepared zirconium oxide containing clove and cardamom extract. After that, it was centrifuged at a high speed. It was centrifuged using Lark refrigerated centrifuge for 10 min at 10,000 rpm. After that, the zirconium oxide nanoparticle pellets were collected for antimicrobial, anti-inflammatory and cytotoxicity testing.

### Characterization of synthesized Zro2 Nanoparticles:

**Figure 3:**
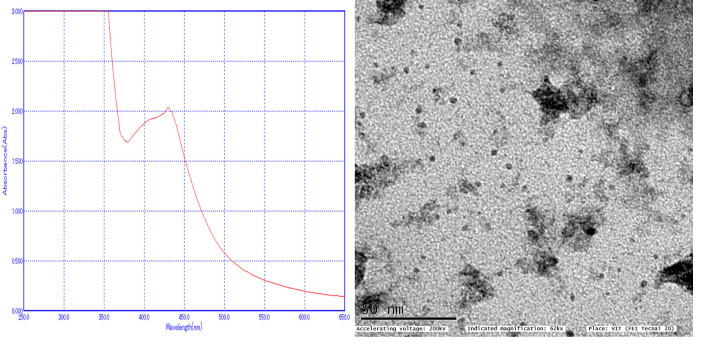
UV-vis spectroscopy: SPR peak at 430 nm; Right: TEM analysis, 5-20 nm spherical nanoparticle.

UV-vis spectroscopy is used to characterize the nanoparticles solution and recorded the UV-vis absorption peak of the ZrO2 NP's. 3 mL of solution was placed in a cuvette and scanned in a double beam UV-visible spectrophotometer (ELICO SL 210 UVVis spectrophotometer) from 300 to 700 nm wavelengths and results are graphically recorded. The size and shape of the nanoparticles were obtained using Transmission electron microscopic analysis (TEM) (Figure 3).

### Antimicrobial activity of Zro2 Nanoparticles:

**Figure 4:**
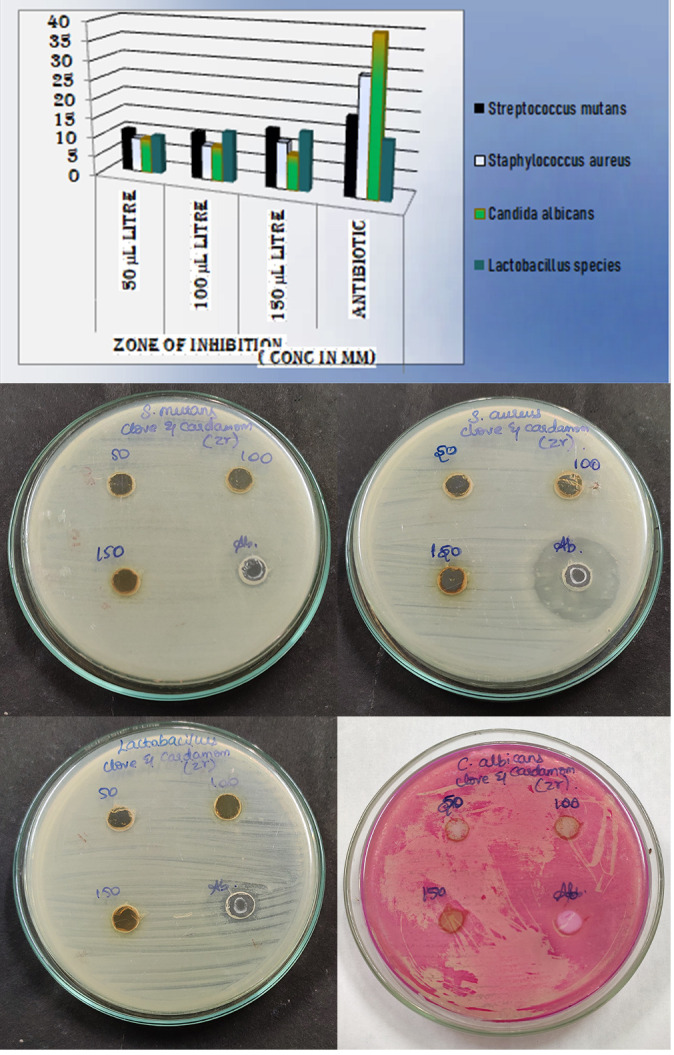
depicts the various zones of inhibition for the ZrO2 NP's at different concentrations of the nanoparticles for different oral pathogens (Streptococcus mutans, Staphylococcus aureus, Lactobacillus species, Candida albicans)

The antimicrobial efficiency of Zirconium oxide nanoparticles was assessed using the agar well diffusion method. Mueller Hinton agar was prepared, sterilized in an autoclave for 15-20 minutes at 121 degrees Celsius and allowed to solidify on sterile petri plates. Streptococcus mutans, Staphylococcus aureus, and Lactobacillus species were swabbed with sterile cotton buds after solidification. A T-shaped well cutter was used to create the wells. Among four wells per plate 3 wells were loaded with clove-cardamom zirconium oxide nanoparticles solution in the concentration of 50 µl, 100 µl, 150 µl and fourth well loaded with a standard antibiotic (Amoxyrite) and then incubated at 37°C for 24 hours. Rose Bengal Agar was prepared as the medium for Candida albicans and inoculated plates were incubated at 37°C for 48 hours. After incubation, the plates were observed and measured for zones of inhibition around the nanoparticle loaded wells (Figure 4).

### Cytotoxic Effect:

**Figure 5:**
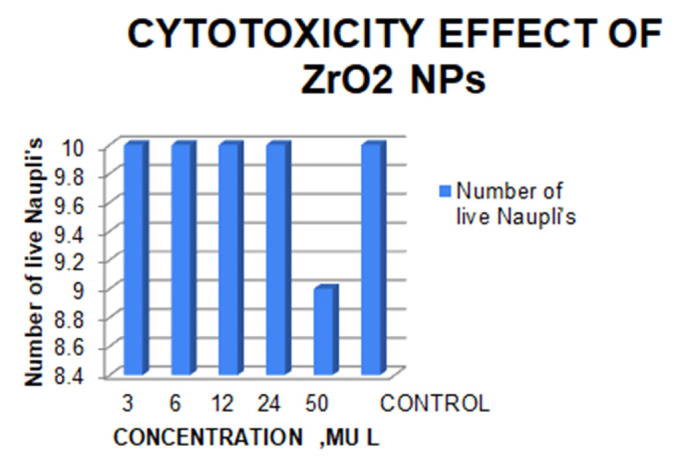
Cytotoxicity - Brine Shrimp lethality assay of clove and cardamom mediated ZrO2 nanoparticles.

Setup preparation: 6L of distilled water was added to the artemia tank. 50 g of iodine-free salt was mixed. 2 capsules of 15 g of Brine Shrimp eggs were placed in the tank and left undisturbed for 5 minutes to allow for proper soaking in salt water. The aeration level was increased to the maximum level with the help of airline tip. After 24 hrs of incubation, nauplii hatch out of the brine shrimp eggs and they're examined under a stereomicroscope. The Brine shrimp assay was used to evaluate the cytotoxicity of ZrO2 nanoparticles reinforced with clove and cardamom extract. 10 nauplii were added to each twelve well of ELISA plates filled with 6-8 ml of saltwater. Each well received varying quantities of ZrO2 nanoparticles augmented with clove and cardamom (3 µL, 6 µL, 12 µL, 24 µL, 50 µL). The total number of live and dead nauplii was counted after incubation of 24 hours and the mortality rate was calculated (Figure 5).

### Anti-Inflammatory Activity (Protein Denaturation Assay):

The activity is done using Bovine Serum Albumin reagent (BSA), which makes 60% of all proteins in animal serum. When BSA is heated, it becomes denatured and begins to exhibit antigens linked to Type III hypersensitivity reaction, which are related to a disease such as rheumatoid arthritis, glomerulonephritis, serum sickness, and systemic lupus erythematosus. 2 mL of 1% bovine albumin was mixed with 400 µl of plant crude extract at various concentrations (500-100 g/mL) at pH 6.8 with 1N HCl and incubated at 37°C for 20 minutes followed by heated in a water bath at 55°C for 20 minutes, then cooled to a room temperature. At 660 nm absorbance value was recorded. An equal amount of ZrO2 nanoparticles reinforced with clove and cardamom was replaced with dimethyl sulfoxide for control. Different concentrations of diclofenac sodium were used as standards. The experiment was performed in triplicate.

### Test Group:

In 5 test tubes, 10 µL, 20 µL, 30 µL, 40 µL and 50 µL of the ZrO2 nanoparticles reinforced with clove and cardamom were taken, respectively. 2 mL of 1 percentage Bovine Serum Albumin (BSA) was added to each test tube. 390 µL, 380 µL, 370 µL, 360 µL and 350 µL of distilled water was added to the test tube comprising 10 µL, 20 µL, 30 µL, 40 µL and 50 µL of ZrO2 nanoparticles respectively.

### Control Group:

To 2 mL of BSA solution, 2 mL of Dimethyl Sulphoxide (DMSO) was added.

### Standard Group:

**Figure 6:**
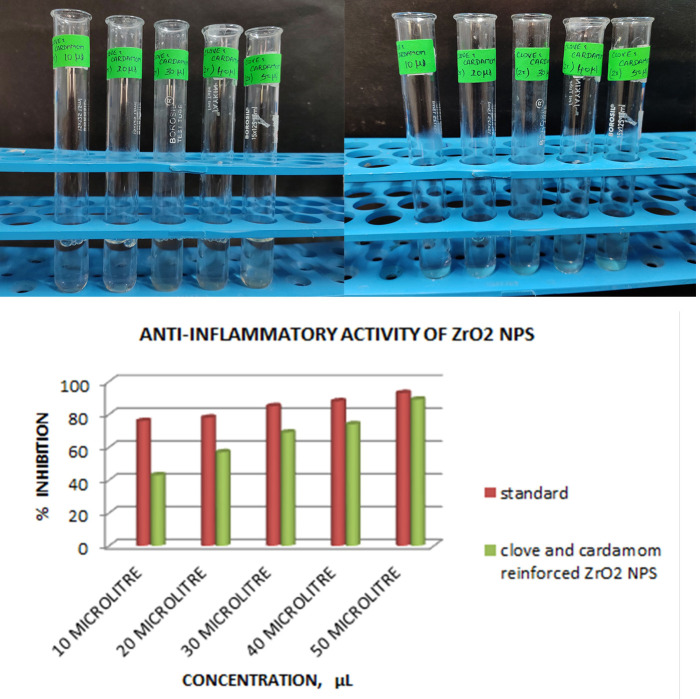
A (left to right) depicts the anti- inflammatory property of ZrO2 nanoparticles reinforced with clove and cardamom extract pre-incubation and post-incubation (Colour change). B depicts the anti- inflammatory property of ZrO2 nanoparticles reinforced with clove and cardamom at various concentrations compared with the standard values.

In 5 test tubes, 10 µL, 20 µL, 30 µL, 40 µL and 50 µL of Diclofenac Sodium were taken respectively. 2 mL of 1 percent Bovine Serum Albumin (BSA) was added to each test tube followed by incubation for 10 minutes at room temperature. Finally, they were incubated for 10 minutes in a water bath at 55°C and absorbance was recorded at 660 nm (Figure 6).

The following formula was used to compute % Inhibition: (see PDF)

### Method of coating on orthodontic arch wires such as NiTi and SS by using Digital magnetic stirrer with hot plate:

**Figure 7:**
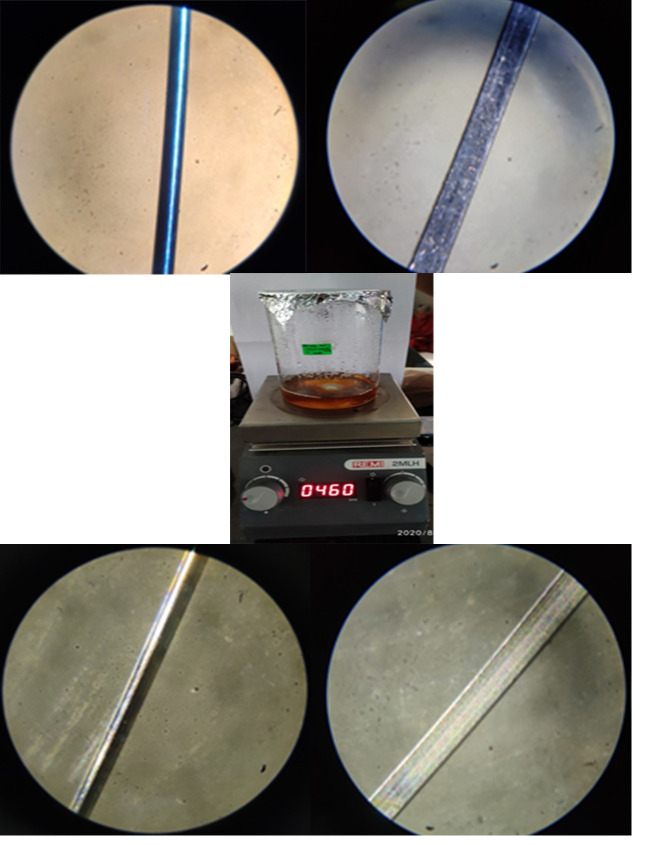
Method of coating on orthodontic arch wires such as NiTi and SS by using Digital magnetic stirrer with hot plate. (From left to right) (A) Uncoated NiTi orthodontic wire, (B) uncoated SS orthodontic wire, (C) zirconium oxide nanoparticles solution with orthodontic arch wire placed in a magnetic stirrer, (D) coated NiTi orthodontic wire; (E)coated SS orthodontic wire.

A magnetic stirrer is made up of a magnetic bar that is inserted into the liquid and stirs it. Another spinning magnet or set of electromagnets in the stirrer unit, underneath the vessel containing the liquid, drives the stir bar's motion. Stir bars are usually coated with Polytetrafluoroethylene (PTFE) or less frequently with glass; the coatings are formulated to be biocompatible, ensuring they won't contaminate or interfere with the reaction mixture they are within. Magnetic stirrers are bar-shaped with a cross-section that is commonly octagonal or circular. Many stir bars have a pivot ring around the core on which they rotate, and they are available in millimeter and centimeter sizes and it can be easily cleaned and sterilized. It has a heating element that can range in power from a few hundred to thousand watts. The surface of the retrieved orthodontic archwires, such as 0.014 NiTi and 19X25 SS of each two, was examined under an optical microscope with a magnification power of 100X to evaluate for roughness and cracks on the surface. It is then placed in a beaker containing a solution of ZrO2 nanoparticles and covered with aluminium foil. Finally, it was placed in a magnetic stirrer and the temperature was set to 450°C to 470°C. It was continuously stirred for about 15 days to allow for a consistent coating process until the solution vaporised and the archwires were coated. As soon as the solution had dried up the colour change was visibly observed. The coated wire was then examined under a microscope to capture the surface of archwire coatings (Figure 7).

## Results:

### Synthesis of ZrO2 NPs:

The clove and cardamom extract when mixed with ZrO2 solution showed a colour change from light orange red colored to dark orange red colored solution. This colour change indicated the formation of ZrO2NP's (Figure 1&Figure 2).

### UV-vis spectroscopy:

UV-VIS spectroscopic analysis, which showed a peak at 430 nm of visible spectrum by reduction of zirconium, salts to ZrO2 NP's (Figure 3).

Transmission electron microscope: TEM analysis revealed spherical shape of ZrO2 NPs with a size range from 5-20 nm and spherical in shape (Figure 3) which was similar to the results of the study [33].

### Antimicrobial activity of ZrO2 NP's:

The inhibition zones for AuNP’s are at various concentrations for each of these organisms - S. aureus, S. mutans, Lactobacillus species and C. Albicans (Figure 4). ZrO2 NP's showed excellent antimicrobial activity as antibiotic potential against Lactobacillus sp. Good antimicrobial activity have observed against streptococcus mutans and Staphylococcus aureus with inhibition zones of 11 to 15mm and 9 to 12 mm. Candida albicans, which revealed moderate antimicrobial activity with same amount of inhibition zone (9mm) at all nanoparticle concentrations.

### Cytotoxicity of clove and cardamom reinforced ZrO2 NPS:

Table 1(see PDF) represents the cytotoxicity of ZrO2 nanoparticles reinforced with clove and cardamom extract. There was no death of nauplii at concentrations of 3L, 6L, 12L, and 24L, but there was a death of 10% of nauplii at 50L. The cytotoxicity of the nanoparticles increased as the concentration was increased.

### Anti- inflammatory property of ZrO2 nanoparticles reinforced with clove and cardamom:

At 10µL, 20µL, 30µL, 40µL, 50 µL concentrations, the anti-inflammatory properties of nanoparticles were found to be closer to the standard values, indicating that they have good anti-inflammatory activity.

### Method of coating on orthodontic arch wires such as NiTi and SS by using Digital magnetic stirrer with hot plate:

Method of coating on orthodontic arch wires such as NiTi and SS by using Digital magnetic stirrer with hot plate (Figure 7): Under a microscope, the digital magnetic stirrer method of coating on orthodontic archwires such as NiTi and SS revealed a colour shift from silver to orange red, as well as a uniform surface of coatings on wires.

## Discussion:

The synthesis of nanoparticles has progressed quickly especially for medical purposes [34]. Green nanotechnology is considered as an evolving eco-friendly alternative to earlier physio-chemical methods that need toxic chemicals for stability. Other distinct advantage of green chemistry approach is it's comparatively cost efficient [35]. To the best of our knowledge; this is the first study to demonstrate the effectiveness of nano zirconium coating on orthodontic archwires using a digital magnetic stirrer with a hotplate. Any novel methods of nano-synthesis have to necessarily undergo stringent tests to validate its cytotoxicity and characterization test to justify the robustness of its physical properties. Clove and cardamom were incorporated into zirconium oxide nanoparticles employing green synthesis methods, and their antibacterial, anti-inflammatory, and cytotoxic capabilities were investigated, as well as a novel method of digital magnetic stirrer coating on NiTi and SS orthodontic archwires.

Cytotoxicity of ZrO2 NP's was evaluated by using Brine shrimp assay. In this study the naupliis was subjected at 3µL, 6 µL, 12 µL and 24µL concentration of green synthesized zirconium oxide nanoparticles. The results of the brine shrimp assay demonstrated that all naupliis were able to survive at all concentrations except at 50 µL there was a death of 10% of nauplii. This concluded that the cytotoxicity of the nanoparticles was increased with increase in corresponding concentration levels. This clearly defines the extent of concentration that can be safely administered when utilizing green synthesized nanoparticles. Since the parameter to establish toxicity (death of 50% nauplii) was not breached, it can be coated on orthodontic archwires as it showed less toxicity.

The antimicrobial activity of ZrO2 NP's was assessed using nutrient agar well diffusion method against oral pathogens like S. aureus, S. mutans, Lactobacillus sp. and C. Albicans. To measure antibacterial activity of the ZrO2 NPs, Zone of inhibition test (Kirby-Bauer Test), a qualitative method was used. The antimicrobial potency depends on the size of the zone of inhibition. ZrO2 NP's showed excellent antimicrobial activity as antibiotic potential against Lactobacillus sp. with 10mm, 13mm, 15mm at 50 µL, 100 µL, 150 µL respectively. Excellent antimicrobial activity has been observed against streptococcus mutans and Staphylococcus aureus with inhibition zones of 11 to 15mm and 9 to 12 mm. The antimicrobial activity increased as the corresponding concentration was increased. The exception is Candida albicans, which revealed moderate antimicrobial activity with the same amount of inhibition zone at all nanoparticle concentrations. The addition of clove and cardamom reinforced the anti-inflammatory activity of ZrO2 nano particles. Under a microscope, the digital magnetic stirrer method of coating on orthodontic archwires such as NiTi and SS revealed a colour shift from silver to orange red, as well as a uniform surface of coatings on wires. Based on the results of the study, we concluded that reinforcing zirconium oxide nanoparticles with clove and cardamom has a synergistic impact and can be used instead of commercially available antibacterial and anti-inflammatory drugs.

## Limitations:

The study was conducted in vitro, so it cannot be assumed that the results of cytotoxicity, anti-inflammatory activity and antimicrobial activity could be translated into clinical effectiveness. Further research should be conducted to validate the efficiency of this coating method on orthodontic archwires.

## Inference:

These nanoparticles can be delivered in the form of a mouthwash. In vivo research involving people's acceptability values are also recommended in future studies.

## Conclusion:

Green synthesized zirconium oxide nanoparticles have potent antimicrobial, anti-inflammatory with minimal cytotoxicity for further consideration in nanocoatings on orthodontic archwires such as NiTi and Stainless Steel.
